# Complete chloroplast genome of *Plantago asiatica* and its phylogenetic position in Plantaginaceae

**DOI:** 10.1080/23802359.2022.2073838

**Published:** 2022-05-10

**Authors:** Hao Si, Rui Li, Qingde Zhang, Luxian Liu

**Affiliations:** School of Life Sciences, Key Laboratory of Plant Stress Biology, Henan University, Kaifeng, China

**Keywords:** *Plantago asiatica*, chloroplast genome, genome skimming, phylogeny inference

## Abstract

*Plantago asiatica*, an herbaceous perennial species of Plantaginaceae, has been used as a traditional herbal medicine plant in China. In this study, the complete chloroplast (cp) genome of *P. asiatica* was sequenced and assembled using genome skimming data. The cp genome was 165,045 bp in length including the large single-copy (LSC, 82,964 bp) and small single-copy (SSC, 4,633 bp) regions separated by two copies of inverted region (IR, 38,724 bp). The cp genome encoded 113 unique genes, consisting of 79 protein-coding genes, 30 tRNA genes, and four rRNA genes, additionally with 27 duplicated genes in the IR regions. Phylogenetic analysis indicated that the representative species from *Plantago* was monophyletic and they were divided into four subgenera. *P. asiatica* belongs to the subgenus *Plantago* and was sister to *P. rigida* with high bootstrap value support.

*Plantago* L. is a genus of Plantaginaceae, and there are about 250 annual and perennial herbs and subshrubs distributed worldwide (Ronsted et al. 2002). Several species within the genus have long been used in traditional Chinese medicine with some chemical components that have good therapeutic effects on certain diseases (Lan et al. 2020). *P. asiatica* Linnaeus 1753, is a perennial flowering plant of *Plantago*, which is native to East Asia (China, Japan, Korea, etc.) (Rahn 1996). According to traditional Chinese medicine, all parts of the plant are medicine to cool heat and promote urination, cause diuresis, clear damp-heat, brighten the eyes, and dislodge phlegm (Li et al. 2020). However, the complete chloroplast (cp) genome of *P. asiatica* has not been sequenced, and more data are needed to reveal the phylogenetic relationships of species in Plantaginaceae (Mower et al. 2021). In this study, the complete cp genome of *P. asiatica* was determined using genome skimming data. The genome sequence of *P. asiatica* was deposited into GenBank with the accession number MZ779005.

The whole genomic DNA was isolated from fresh leaf tissue of one *P. asiatica* plant collected in Kaifeng (China; 114°18′37.56″E, 34°49′20.96″N) using Plant DNAzol Reagent (LifeFeng, Shanghai, China) according to the manufacturer’s protocol. A voucher specimen (LLX2021061701; Luxian Liu, liushuangcx2007@126.com) was deposited at the Herbarium of Henan University (HHN) and no specific permissions were required for sample collection which are neither privately owned nor protected and the field study did not involve endangered or protected species. High-quality DNA was sheared and sequenced on an Illumina HiSeq X10 by Beijing Genomics Institute (BGI, Wuhan, China) with 150 bp paired-end reads. The raw Illumina reads were filtered by quality with Phred scores of 30 or less, and all the remaining reads were assembled into contigs implemented in the CLC Genomic Workbench (CLC Inc., Aarhus, Denmark). The complete cp genome of *P. asiatica* was reconstructed and annotated using the software Geneious R11 (Biomatters, Auckland, New Zealand) with *P. australis* Lam. 1792 (GenBank accession number: MW877569) as a reference following description in Liu et al. (2017) and Liu et al. (2020). Phylogenetic tree was inferred based on whole cp genomes of 38 Plantaginaceae species using maximum-likelihood (ML) method with two *Forsythia* L. species as outgroups. ML analysis was implemented in RAxML-HPC v8.2.12 on the CIPRES cluster (Miller et al. 2010), 1000 bootstrap iterations were conducted with other parameters using the default settings and GTR + I+G was determined by the software jModel Test v2.1.4 (Posada 2008) as the best-fit nucleotide substitution model.

We generated 288,424,678 paired-end reads for *P. asiatica*, and 140,664,575 reads were removed from the raw data after trimming low quality sequences. The cp genome of *P. asiatica* was 165,045 bp in length, and shared the common feature of involving a typical quadripartite structure consisting of an 82,964 bp large single-copy region (LSC), a 4633 bp small single-copy region (SSC), and two 38,724 bp inverted repeats (IRs). One hundred and thirteen unique genes were annotated in the cp genome of *P. asiatica*, containing 79 protein-coding genes, 30 tRNA genes, and four rRNA genes with additional 27 duplicated genes in the IR regions. Among them, six tRNA genes and 10 protein-coding genes contained a single intron and two genes (*clp*P and *ycf*3) contained two introns. The overall GC content of the total length, LSC, SSC, and IR regions was 38.1%, 36.7%, 30.7%, and 39.9%, respectively. The phylogeny revealed that all the *Plantago* species formed a monophyletic clade and were divided into four subgenera, which was consistent with the description in Rahn (1996) and Mower (2021), and *P. asiatica* was closest relationship to *P. rigida* Kunth 1818 with high support in the 25 *Plantago* species (Figure 1). Comparative analysis showed that the cp genomes from subg. *Coronopus* and subg. *Plantago* had larger genome size due to the substantial IR expansions that simultaneously reduced the size of the SSC.

**Figure 1.**
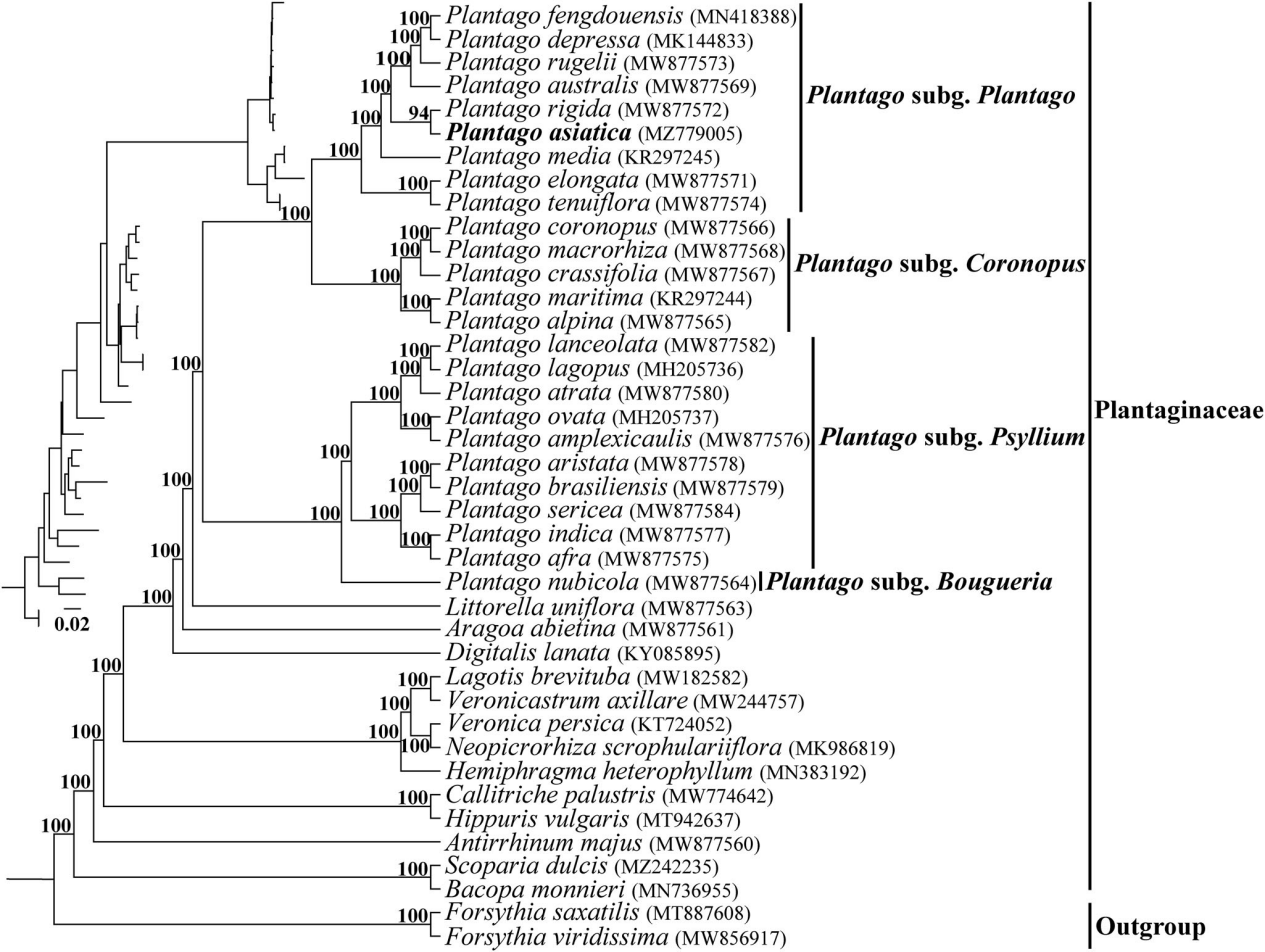
The phylogenetic tree of Plantaginaceae was inferred according to the whole chloroplast genome sequence. The inset topology in the upper left shows the relative branch lengths in substitutions per site. Numbers above the lines represent bootstrap values from maximum-likelihood analyses.

In conclusion, this study revealed the whole cp genome of *P. asiatica* for the first time. It will provide necessary and important genetic resources for better study of phylogeny of *Plantago* in the future.

## Data Availability

The genome sequence data that support the findings of this study are openly available in GenBank of NCBI at https://www.ncbi.nlm.nih.gov/ under the accession no. MZ779005. The associated Bio-Project, SRA and Bio-Sample numbers of the raw sequence data for assembling the cp genome are PRJNA754501, SRR15458684, and SAMN20777470, respectively.
